# Transcriptome Analysis Reveals the Effect of Oyster Mushroom Spherical Virus Infection in *Pleurotus ostreatus*

**DOI:** 10.3390/ijms25179749

**Published:** 2024-09-09

**Authors:** Yifan Wang, Junjie Yan, Guoyue Song, Zhizhong Song, Matthew Shi, Haijing Hu, Lunhe You, Lu Zhang, Jianrui Wang, Yu Liu, Xianhao Cheng, Xiaoyan Zhang

**Affiliations:** 1 School of Agriculture, Ludong University, Yantai 264025, China; 2 Department of Plant Science, University of Cambridge, Cambridge CB2 3EA, UK; 3 Yantai Growth Drivers Conversion Research Institute and Yantai Science and Technology Achievement Transfer and Transformation Demonstration Base, Yantai 264001, China

**Keywords:** oyster mushroom spherical virus, *Pleurotus ostreatus*, transcriptome analysis, carbohydrate-active enzymes, pathogenic mechanism

## Abstract

Oyster mushroom spherical virus (OMSV) is a mycovirus that inhibits mycelial growth, induces malformation symptoms, and decreases the yield of fruiting bodies in *Pleurotus ostreatus*. However, the pathogenic mechanism of OMSV infection in *P. ostreatus* is poorly understood. In this study, RNA sequencing (RNA-seq) was conducted, identifying 354 differentially expressed genes (DEGs) in the mycelium of *P. ostreatus* during OMSV infection. Verifying the RNA-seq data through quantitative real-time polymerase chain reaction on 15 DEGs confirmed the consistency of gene expression trends. Both Gene Ontology and Kyoto Encyclopedia of Genes and Genomes analyses highlighted the pivotal role of primary metabolic pathways in OMSV infection. Additionally, significant changes were noted in the gene expression levels of carbohydrate-active enzymes (CAZymes), which are crucial for providing the carbohydrates needed for fungal growth, development, and reproduction by degrading renewable lignocellulose. The activities of carboxymethyl cellulase, laccase, and amylase decreased, whereas chitinase activity increased, suggesting a potential mechanism by which OMSV influenced mycelial growth through modulating CAZyme activities. Therefore, this study provided insights into the pathogenic mechanisms triggered by OMSV in *P. ostreatus*.

## 1. Introduction

Mycoviruses are ubiquitously distributed across all major fungal taxa and were discovered later than viruses infecting plants, animals, and prokaryotes [1,2,3]. In 1962, the first fungal virus, La France isometric virus, was discovered to infect the economically important *Agaricus bisporus* [4]. To date, more than 60 distinct mycoviruses have been documented to infect edible fungi, encompassing double-stranded RNA (dsRNA), positive-sense single-stranded RNA (+ssRNA), and negative-sense single-stranded RNA (−ssRNA) viruses [5,6]. Most mycoviruses are latent or asymptomatic in edible fungi hosts, whereas some have deleterious effects, such as altering mycelial growth, colony morphology, sporulation, coloration, and virulence [7,8,9,10].

Virus infection involves an interaction between the virus and the host. During pathogenesis, transcriptional analysis can be performed to focus on the pathogenic strategy of the virus. RNA sequencing (RNA-seq) provides a novel approach to transcriptome analysis and is widely used to reveal the response of fungal hosts to mycoviral infection [11]. During the ssRNA mycovirus *Cryphonectria* hypovirus 1 (CHV1) infection, significant, yet specific, changes in primary and secondary metabolism occurred, and antiviral fungal metabolites were induced in *Cryphonectria parasitica* [12]. During *Beauveria bassiana* chrysovirus 2 infection in host fungi *B. bassiana*, transcriptome analysis showed that genes related to mycelial growth, insect epidermis penetration, and toxin metabolism were downregulated [13]. Recent data have shown that *Bipolaris maydis* partitivirus 36 (BmPV36) curtails the virulence of *B. maydis* by dismantling host cellular architecture, suppressing toxin and cell wall-degrading enzyme synthesis, and decelerating cellular metabolism [14]. The transcriptomic profiling of mushroom virus X (MVX) in *Agaricus bisporus* implied that the host attempted to curtail infection by dampening vesicular transport activities [15]. Despite these insights, exploration into mycoviruses infecting mushrooms remains limited.

Carbohydrate-active enzymes (CAZymes) are for hydrolyzing plant cell wall polysaccharides and are essential for substrate degradation processes [16]. Fungal CAZymes are categorized into five main families: glycoside hydrolase (GH), glycosyltransferase (GT), polysaccharide lyase (PL), carbohydrate esterase (CE), and auxiliary activities (AA) [17]. The CAZy database classifies laccase within the AA family, which is involved in lignin degradation. Cellulase, chitinase, and amylase belong to the GH family, and are responsible for degrading cellulose, chitin, and starch, respectively. Additionally, the activity levels of these enzymes can be influenced by various factors, including environmental conditions and the presence of pathogens or symbionts, such as mycoviruses. Several studies have demonstrated that mycovirus infection can alter the activities of host CAZymes. Laccase activities were significantly reduced in the dsRNA virus-infected *C. parasitica* [18]. Similarly, BmPV36 infection significantly decreased the expression of genes involved in the synthesis of cellulase, pectinesterase, and cutinase in *B. maydis* [14]. In addition, the dsRNA mycovirus *Pleurotus ostreatus* virus-ASI2792 influenced spawn growth and fruiting body development in *P. ostreatus* by reducing the expression and activities of certain CAZymes, including amylase and chitinase [19].

*P. ostreatus*, a globally cultivated edible mushroom, has high economic, nutritional, and therapeutic significance [20,21]. The oyster mushroom can be infected by several dsRNA and ssRNA mycoviruses [22,23,24,25,26,27]. The oyster mushroom spherical virus (OMSV), the first ssRNA virus identified in *P. ostreatus*, causes mushroom dieback disease. OMSV is a spherical virus, 27 nm in diameter, with a genome of 5784 bp encoding seven open-reading frames [28]. It has been detected in multiple regions in China, including Beijing City, Shandong Province, and Jilin Province [29,30,31]. In previous studies, an OMSV−China strain was isolated from *P. ostreatus* 8129, which could be transmitted horizontally to virus-free *Pleurotus pulmonarius* or *Pleurotus floridanus* strains and vertically propagated via basidiospores [32,33]. The OMSV infection caused mycelium retardation and fruiting body deformation, leading to reduced mushroom yields and a decline in their commercial value [31]. However, studies exploring the pathogenic mechanisms underlying OMSV infection remain scarce. Therefore, identifying and analyzing the host factors involved in OMSV infection and elucidating the OMSV pathogenic mechanism are essential.

In this study, RNA-seq was used for the first time to investigate the response of *P. ostreatus* to OMSV infection. Also, the activities of extracellular enzymes, including laccase, carboxymethyl cellulase (CMCase), amylase, and chitinase, were also analyzed during OMSV infection in *P. ostreatus* to examine the relationship between OMSV infection and the phenotypic changes it induced in *P. ostreatus*. This study offered a theoretical basis for deeper exploration of the pathogenic mechanisms of OMSV in *P. ostreatus*, thus assisting in developing disease-resistant varieties of edible fungi and preventing and controlling diseases.

## 2. Results

### 2.1. Transcriptome Data Assembly Results

**Figure 1:**
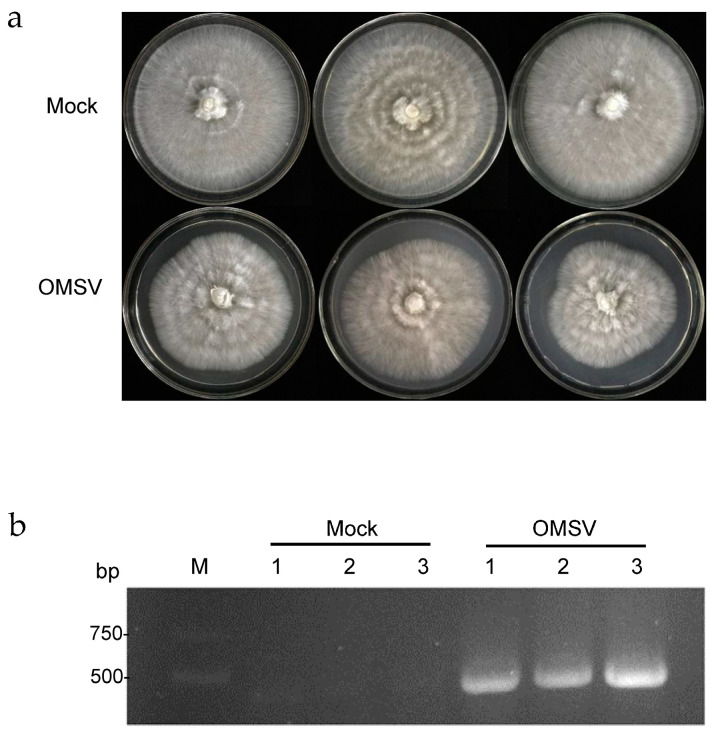
The mycelium growth of OMSV-free (Mock) and OMSV−infected (OMSV) *P. ostreatus* strains. (**a**) The mycelium growth on PDA plates after seven days of cultivation. (**b**) RT-PCR detection of OMSV. Lane M, DNA Marker2000. Numbers 1–3 represent three biological replicates.

Previous studies revealed that OMSV inhibited mycelial growth and induced deformities in the fruiting bodies of *P. ostreatus* [31,32]. The OMSV-free and OMSV-infected mycelia of strain *P. ostreatus* 8129 were collected for transcriptome sequencing to identify the genes associated with OMSV infection in *P. ostreatus*. As shown in Figure 1a, a significant decrease in the growth rate of OMSV−infected (OMSV) mycelia was observed compared with that of OMSV-free (Mock) mycelia of *P. ostreatus* after 7 days of inoculation. This was followed by reverse transcription–polymerase chain reaction (PCR) detection (Figure 1b). Two cDNA libraries, OMSV-free and OMSV-infected strains, were sequenced, and 41.21 Gb of clean data were obtained. The samples with Q30 above 92.63% (exceeding 90%) indicated high data quality. Additionally, the GC content above 50% indicated that the sequencing was not obviously biased (Table 1). The data quality was considered satisfactory, indicating that the sequencing data could be further analyzed for its quality and accuracy.

### 2.2. Expression Analysis

**Figure 2:**
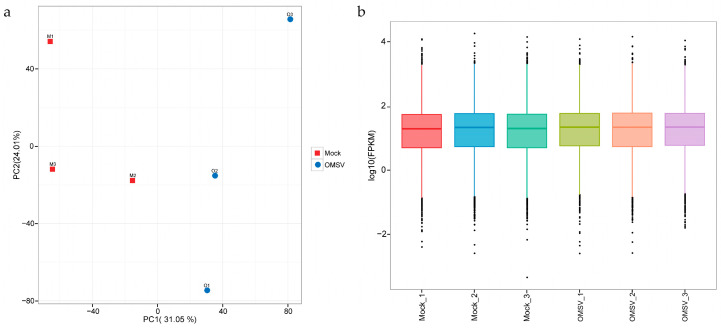
Gene expression analysis of OMSV-free (Mock) and OMSV-infected (OMSV) strains of *P. ostreatus*. (**a**) Principal component analysis (PCA) of each sample. The FPKM values of each sample were used to perform PCA. (**b**) The FPKM box plots of each sample. The horizontal axis represents the sample names, while the vertical axis displays the log10 (FPKM) values. Different colors denote distinct samples.

As depicted in Figure 2a, principal component 1 (PC1) accounted for 31.05% of the sample variability, whereas principal component 2 (PC2) accounted for 24.01%. The distribution of the highest, lowest, and median log10 fragments per kilobase of transcript per million mapped reads (FPKM) values of gene expression across different samples is displayed in a boxplot in Figure 2b. The results demonstrated that the gene expression in *P. ostreatus* was relatively concentrated, with the median log10 (FPKM) values for both the OMSV-infected and OMSV−free strains clustering around 1.

### 2.3. Differential Gene Analysis

**Figure 3:**
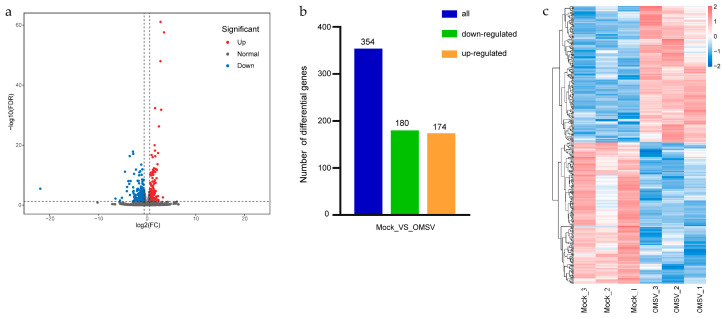
Differential gene analysis of OMSV-free (Mock) and OMSV-infected (OMSV) *P. ostreatus* strains. (**a**) Volcano plot of all identified genes. Genes with upregulated expression are indicated by red dots, those with downregulated expression by blue dots, and genes not differentially expressed by gray dots. (**b**) The statistical map of DEGs. The horizontal axis indicates the comparison names, and the vertical axis indicates the number of DEGs. (**c**) Heatmap displaying the clustering analysis of all DEGs for the OMSV-free and OMSV-infected strains of *P. ostreatus*. The expression levels of DEGs were normalized using the log10 FPKM method. Each row represents a single gene, and each column corresponds to a sample group. The color gradient from blue to red indicates that the FPKM value ranges from low to high.

Differentially expressed genes (DEGs) in OMSV infection were identified using a false discovery rate (FDR) corrected *p*-value ≤ 0.05 and a fold change ≥ 1.5. The sequencing results revealed 354 DEGs on comparing the OMSV−infected strain with the OMSV-free strain (Figure 3a). Among these 354 DEGs, 174 were upregulated and 180 were downregulated (Figure 3b). The heat map of the hierarchical cluster analysis further supported these results, confirming the difference between the two sample groups (Figure 3c).

### 2.4. KOG Functional Enrichment Analysis of DEGs

**Figure 4:**
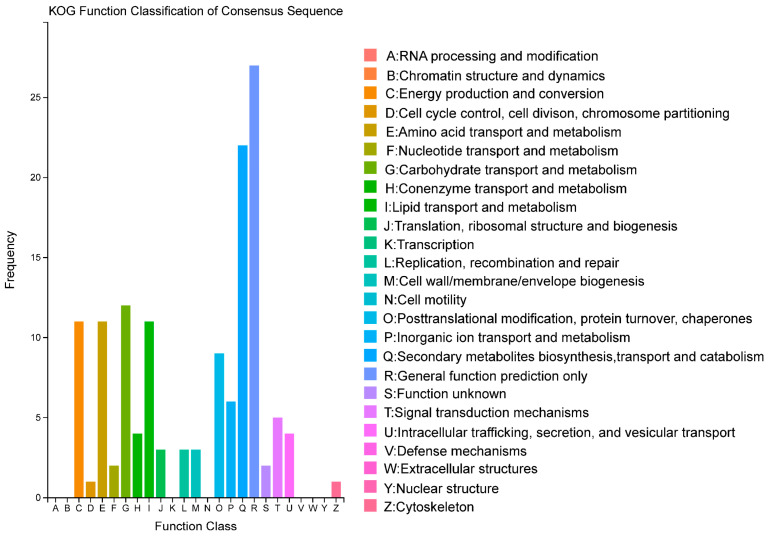
KOG analysis of the DEGs of OMSV-free and OMSV-infected *P. ostreatus* strains. The vertical axis indicates the number of DEGs within a specific functional cluster, while the horizontal axis represents the functional classes.

Sequences with Basic Local Alignment Search Tool (BLAST) software (version 2.2.26) hits were further classified through Eukaryotic Ortholog Group (KOG) pathway analysis. This analysis revealed that 134 DEGs were annotated and enriched into 19 functional classifications. Also, the DEGs were mainly distributed in the cluster related to “secondary metabolites biosynthesis, transport and catabolism, energy production and conversion, carbohydrate transport and metabolism, amino acid transport and metabolism, and lipid transport and metabolism”, all of which contained higher-than-average amounts of DEGs. The cluster of “secondary metabolites biosynthesis, transport and catabolism” accounted for 16% of the total annotated DEGs (Figure 4). Lectin is a protein that binds to carbohydrates and displays a wide range of biological properties, including antimicrobial, antifungal, and antiviral activities [34]. The transcriptome data analysis revealed that one gene-encoding lectin (g1097844) was significantly upregulated and belonged to the protein encoded by the ricin-type β-trefoil lectin domain.

### 2.5. Gene Ontology and Kyoto Encyclopedia of Genes and Genomes Functional Enrichment Analysis of DEGs

**Figure 5:**
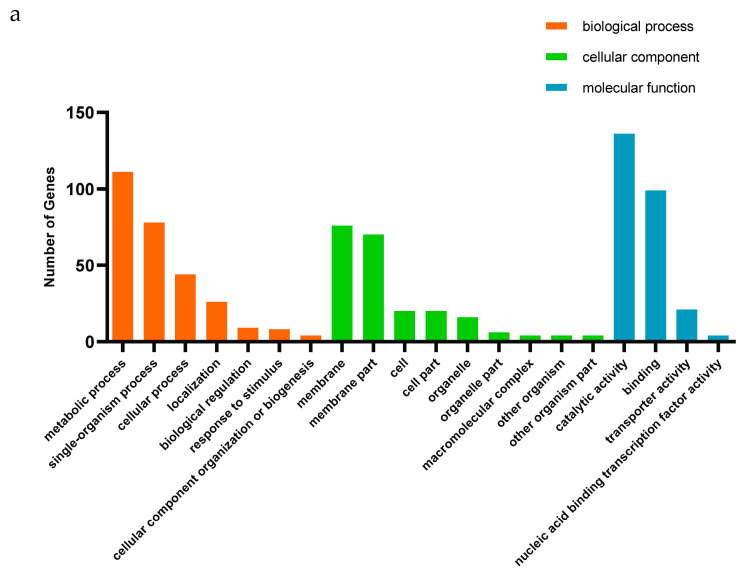

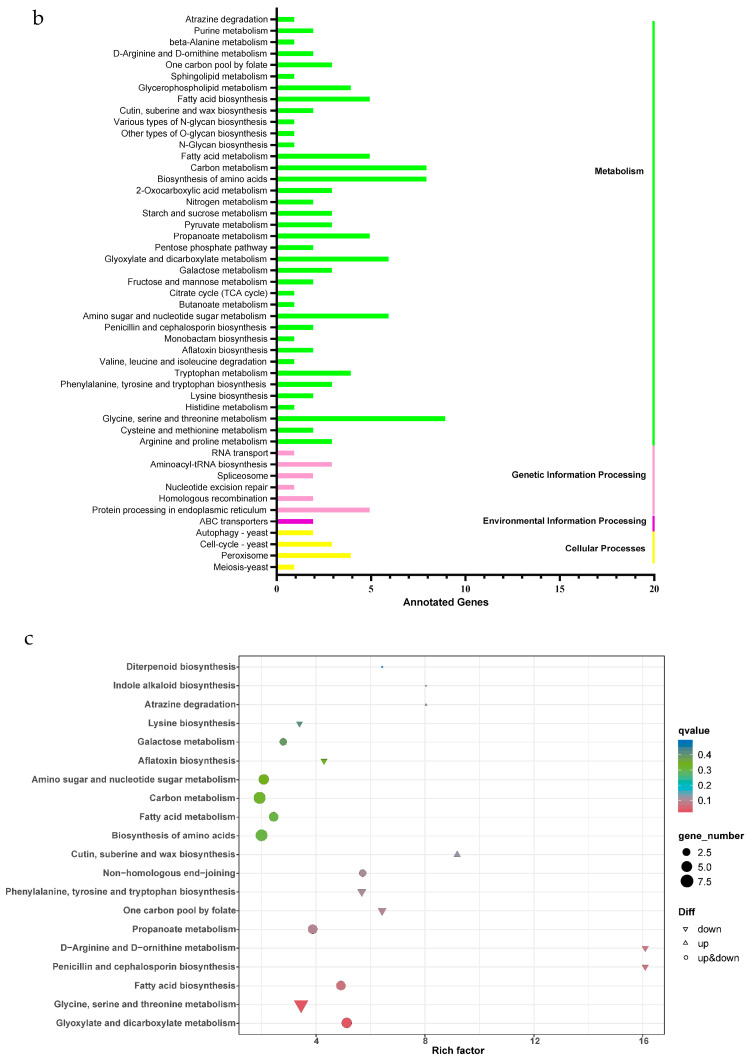
GO and KEGG function classification of DEGs of OMSV-free and OMSV-infected *P. ostreatus* strains. (**a**) GO pathway-annotated genes. The vertical axis represented the name of the enriched GO term, while the horizontal axis indicated the number of DEGs within the corresponding term. Different colors represent three different categories. (**b**) KEGG pathway-annotated genes. Different colors represent four different categories. (**c**) Statistics of KEGG enrichment.

The 230 DEGs annotated in the Gene Ontology (GO) database were dispersed across 30 functional categories, including 12 subclasses in biological process (BP), 11 subclasses in cell component (CC), and 7 subclasses in molecular function (MF). Among these 230 DEGs, 119 were upregulated and 111 were downregulated. In the BP branch, the three subcategories containing the most DEGs were metabolic processes, single-organism processes, and cellular processes with 111, 78, and 44 DEGs, respectively. In the CC branch, the 2 subclasses containing the most DEGs were membrane and membrane part, with 76 and 70 DEGs, respectively. In the MF branch, the 3 subclasses with the most DEGs were catalytic, binding, and transporter activities with 136, 99, and 21 DEGs, respectively (Figure 5a). GO functional enrichment analysis was performed to explore the infection mechanism of OMSV at the molecular level. This analysis was conducted for both upregulated and downregulated DEGs, yielding distinct and significant results. A significant enrichment of upregulated DEGs was noted in primary metabolism, including carbohydrate metabolic processes (GO: 0005975), oxidoreductase activity (GO: 0016491), and hydrolase activity (GO: 0004553), which are crucial for survival during viral infection. Moreover, the downregulated DEGs were mostly enriched in all three branches, including an integral component of the aromatic amino acid family biosynthetic process (GO: 0009073), heme binding (GO: 0020037), iron ion binding (GO: 0005506), monooxygenase activity (GO: 0004497), and membrane (GO: 0016021).

Kyoto Encyclopedia of Genes and Genomes (KEGG) pathway enrichment analysis indicated that 141 DEGs were mainly manifested in four pathways: “Cellular Processes” (10 DEGs), “Environmental Information Processing” (2 DEGs), “Genetic Information Processing” (16 DEGs), and “Metabolism” (113 DEGs). Among these, the number of DEGs related to glycine, serine, and threonine metabolism was the highest (Figure 5b). DEGs of OMSV-infected samples were annotated to 10 KEGG pathways (*p*-value < 0.05) (Figure 5c). The top three KEGG pathways with the largest numbers of DEGs were “glyoxylate and dicarboxylate metabolism”, “glycine, serine, and threonine metabolism”, and “fatty acid biosynthesis” (Table 2), indicating that the carbohydrate metabolism, amino acid metabolism, and lipid metabolism pathways in *P. ostreatus* were affected by OMSV infection. GO and KEGG analysis showed that OMSV infection significantly changed the primary metabolism in *P. ostreatus*.

### 2.6. Quantitative Reverse Transcription-PCR Validation of DEGs

CAZymes play a crucial role in the metabolism of glycoconjugates, polysaccharides, and oligosaccharides. Fungal CAZymes are categorized into five major families: GH, GT, PL, CE, and AA [17]. The investigation of CAZyme gene expression showed that 23 DEGs were related to CAZymes. Of these, 19 were GH family genes, with 15 upregulated (g1057808, g1081432, g35611, g1074848, g1035754, g1093467, g1046178, g696, g21100, g1035175, g49423, g1035282, g1098006, g1088664, and g53126) and 4 downregulated (g152719, g1077485, g160022, and g1103527) genes. The expression of one AA family gene (g51725) and one GT family gene (g1067151) was downregulated, and the expression of one PL family gene (g1036297) and one CE family gene (g1044335) was upregulated.

**Figure 6:**
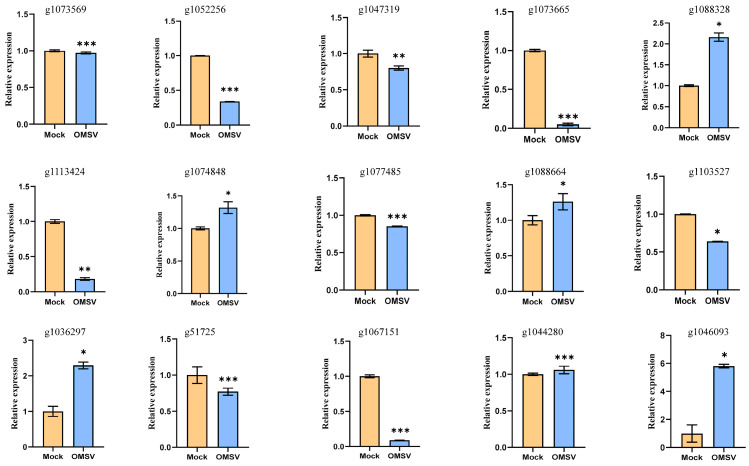
Validation of selected DEGs of OMSV-free (Mock) and OMSV−infected (OMSV) strains of *P. ostreatus* using qRT-PCR. The statistical analysis included a one-way analysis of variance (ANOVA) and *t*-tests, * *p* < 0.05, ** *p* < 0.01, and *** *p* < 0.001.

A total of 15 genes with high expression levels were selected for quantitative reverse transcription-PCR (qRT-PCR) verification, of which 7 belonged to genes encoding carbohydrate enzymes, to validate the RNA-seq results (Table 3). The qRT-PCR results showed that three genes (g1074848, g1088664, and g1036297) were upregulated and four genes (g1077485, g1103527, g1067151, and g51725) were downregulated, which were consistent with the results of RNA-seq. In addition, eight genes (g1073569, g1052256, g1047319, g1073665, g1088328, g1113424, g1044280, and g1046093) with high expression levels were randomly selected for qRT-PCR verification, and their results also aligned with those from RNA-seq (Figure 6).

### 2.7. Effect of OMSV Infection on CAZymes

**Figure 7:**
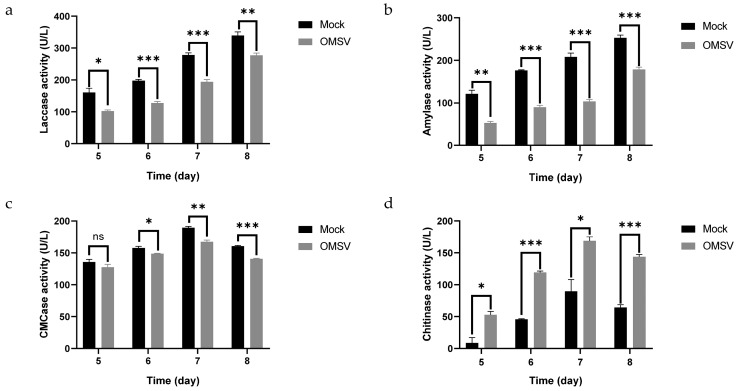
The enzyme activity of OMSV-free (Mock) and OMSV−infected (OMSV) *P. ostreatus* strains during mycelial growth. (**a**) Laccase activity; (**b**) amylase activity; (**c**) CMCase activity; (**d**) chitinase activity. Numbers 5–8 denote the days of mycelial growth in liquid medium. The statistical analysis included a one-way analysis of variance (ANOVA) and *t*-tests, * *p* < 0.05, ** *p* < 0.01, and *** *p* < 0.001.

As a member of the white-rot fungi, *P. ostreatus* uses CAZymes, including laccase, amylase, CMCase, and chitinase, to degrade lignocellulose and provide nutrients essential for mycelial growth [19]. The activities of laccase, chitinase, amylase, and CMCase were measured to investigate the impact on carbohydrate enzymes of OMSV infection. The enzyme activity results showed a gradual rise in laccase and amylase from 5 to 8 dpi, whereas the activities of CMCase and chitinase initially increased before declining, peaking on the seventh day of cultivation (Figure 7). OMSV-infected strains exhibited diminished activities of laccase, CMCase, and amylase compared with OMSV-free stains, along with a marked increase in chitinase activity. These findings suggest that the OMSV infection influenced the physiological responses of *P. ostreatus*, including the activities of CAZymes.

## 3. Discussion

Most mycoviruses are latent in their host fungi; however, some have been shown to affect the growth, pigmentation, and virulence of the host [7,9,35]. OMSV has been reported as a difficult-to-control pathogen responsible for causing oyster mushroom dieback disease [28]. A previous study found that OMSV induced not only retarded mycelial growth but also abnormal fruiting body development, resulting in significant declines in mushroom yield [31]. The present study was novel in describing the pathogenic mechanism of OMSV infection in *P. ostreatus* using transcriptomics. GO enrichment analysis showed that DEGs were significantly enriched in oxidoreductase activity, amino acid metabolism, and heme binding. Heme binding refers to the iron-complexed compound in the porphyrin (tetrapyrrole) ring. The heme assimilation pathway has been identified as a vital iron acquisition method in fungi [36,37,38]. Heme represents a paramount source of iron crucial for microorganisms, and iron is indispensable to nearly all living organisms due to its vital role in metabolism and energy generation [39]. During OMSV infection, significant enrichment of DEGs in the heme-binding pathway was observed. It was hypothesized that OMSV might influence the defense response of *P. ostreatus* by affecting iron formation, a defense strategy previously reported in *Cordyceps militaris* against *Calcarisporium cordycipiticola* [40]. Furthermore, OMSV infection significantly altered the primary metabolism of the host *P. ostreatus*, as evidenced by the enrichment of KEGG pathways linked to carbohydrate, fatty acid, and amino acid metabolism. Previous studies have shown that *Rhizoctonia* infection affects the primary metabolism of soybean seedlings, particularly through carbohydrate, amino acid, and carboxylic acid metabolisms [41]. Similarly, the infection of *C. cordycipiticola* in *C. militaris* significantly changed primary metabolism, including amino acid and 2-oxocarboxylic acid pathways [40].

The fungi can produce various secondary metabolites (such as terpenoids), lectins, and other bioactive substances as part of their chemical defense against viral infection [42,43,44]. Previous reports indicated that enrichment of secondary metabolite pathways plays a crucial role in *Sclerotinia sclerotiorum* defense against *Sclerotinia sclerotiorum* hypovirulence-associated DNA virus 1 (SsHADV-1) infection [45]. A recent study revealed changes in most secondary metabolites of *C. parasitica*, including alkaloids, terpenoids, and polyketides, which might serve as a barrier defense against CHV1 infection. Another study showed that *C. parasitica* defended against CHV1 infection by the changes in most secondary metabolites, including alkaloids, terpenoids, and polyketides [12]. The present study revealed significant enrichment of genes regulating secondary metabolites in the KOG pathways during OMSV infection. Edible mushrooms contain high amounts of lectins, which are carbohydrate-binding proteins of non-immune origin with a specific binding affinity for glycoconjugates [46,47,48,49,50]. Certain lectins are known for their roles in defense responses, although their characterization of these defenses remains limited [51,52]. The lectins isolated from *Gymnopilus* mushrooms were shown to inhibit the growth of *Staphylococcus aureus* and *Aspergillus niger*, indicating the role of lectins in defense responses [34,53]. In addition, recent studies have shown that the lectin gene *Polec2* of *P. ostreatus* plays a role in defense against the mite predator *Tyrophagus putrescentiae* [54]. The present study demonstrated a pronounced elevation in the expression of lectin genes during OMSV infection, suggesting that lectins might be actively involved in the defense response against OMSV, a hypothesis that warrants further investigation.

*P. ostreatus* secretes various extracellular enzymes, including CAZymes, during growth and development. CAZymes can decompose natural macromolecules such as polysaccharides, proteins, and nucleic acids into small molecules that are easily absorbed by edible fungi hyphae and fruiting bodies, providing nutrients for mycelial growth, primordium formation, and fruiting body development. Therefore, these enzymes are essential for achieving optimal mushroom yields [55]. CAZy GH enzymes hydrolyze glycosidic bonds between two or more carbohydrates or between one carbohydrate and non-carbohydrate parts in basidiomycetes, and are key enzymes involved in carbohydrate metabolism [17]. In addition, GHs are common enzymes in nature that degrade the most abundant biomasses, such as cellulose, hemicellulose, and starch [56,57]. The enzymes involved in lignin degradation, such as laccase, which participate in the depolymerization of non-carbohydrate components like lignin, are categorized into AA families [58,59]. The transcriptome data of this study showed that the CMCase gene g152719, amylase gene g160022, and laccase gene g51725 were downregulated during OMSV infection in *P.ostreatus*. The enzyme activity results indicated that the activities of laccase, CMCase, and amylase decreased during OMSV infection, suggesting a potential mechanism by which OMSV influenced mycelial growth through modulating CAZyme activities. The study found that the genes regulating chitinase (g53126, g1057808, g1074848, g696, and g1098006) were significantly upregulated. Chitinase is involved in the defense response against plant pathogens. Studies have shown that the CaChiIII7 chitinase gene in peppers plays a pivotal defensive role against *Colletotrichum acutatum* [60]. Also, a recent study reported that three soybean chitinases (GmChi01, GmChi02, and GmChi16) were involved in defense against *Fusarium oxysporum* [61]. Similarly, the chitinase of *Brassica napus* played a role in defense against *Leptosphaeria maculans* [62]. Based on the upregulation of chitinase gene expression and increased enzyme activity during OMSV infection, it is speculated that chitinase and its active products may serve as signaling molecules, activating the expression of host defense-related genes, which deserves further validation.

## 4. Materials and Methods

### 4.1. RNA Extraction

The mycelium of OMSV-free and OMSV−infected *P. ostreatus* strains were cultivated in a constant temperature incubator at 24 °C for 7 days. Total RNA extraction from *P. ostreatus* mycelia was meticulously conducted utilizing the RNA extraction kit (Tiangen, Beijing, China), adhering strictly to the manufacturer’s protocol. For the detection of RNA purity and concentration, the NanoDrop 2000 spectrophotometer was used. The integrity of RNA is accurately detected using Agilent 2100 (Agilent Technologies, Santa Clara, CA, USA).

### 4.2. Reverse Transcription PCR

Preparation of the 20 μL RT master mixture for the RT reaction consisted of 5 μL of RNA, 4 μL of RT 5× buffer, 0.5 μL of reverse primer (OMSV-R), 0.25 μL of M-MLV reverse transcriptase, 0.25 μL of RNase Inhibitor, 1 μL of dNTP mix, and 9 μL of ddH_2_O. The RT process was carried out at 37 °C for 90 min. After the RT reaction, the PCR mixture was prepared containing 2 μL cDNA template, 10 μL 2 × Taq PCR MasterMix II (Tiangen, Beijing, China), 0.5 μL primers (OMSV-F/OMSV-R, ACCCCCCCAGGATCTCAAGCTTC/GAGATGTAGACRTTGAAAGC) for each, and 7 μL ddH_2_O final volume of 20 μL for PCR amplification. Amplification products were electrophoresis in 1% agarose gel.

### 4.3. cDNA Library Preparation and Illumina Sequencing

Following sample qualification, library construction proceeded, with eukaryotic mRNA enrichment using magnetic beads with Oligo (dT). First-strand cDNA was synthesized using fragmented mRNA as the template and random hexamers as primers. Subsequently, second-strand synthesis was performed using a DNA polymerase I system. The integrity and quality of the generated libraries were meticulously assessed using the Qubit 3.0 Fluorometer, quantitative real-time PCR (qRT-PCR), and the Qsep400 high-throughput analysis system. The cDNA library was sequenced using the Illumina NovaSeq 6000 platform at the Beijing Biomarker Technologies Co., Ltd. (Beijing, China).

### 4.4. Data Processing and Differential Expression Analysis

Raw sequencing reads underwent rigorous quality control, eliminating adapter sequences, reads harboring over 10% unknown nucleotides, and those of low quality, yielding a set of cleaned reads. These cleaned reads were then aligned to the reference genome of *P. ostreatus* utilizing the HISAT2 (version 2.2.0) alignment tool. Transcript abundances were estimated by employing the widely recognized metric of fragments per kilobase of transcript per million mapped reads (FPKM) [63]. Ultimately, we pinpointed DEGs through application of the DESeq2 methodology [64]. Throughout this differential expression analysis, genes with a fold change ≥ 1.5 and an FDR < 0.05 were deemed statistically significant and selected for further investigation.

### 4.5. Functional Analysis of DEGs

To elucidate the functional roles and pathways of the differentially expressed genes (DEGs), comprehensive analyses were conducted, encompassing Eukaryotic Ortholog Groups (KOGs), Gene Ontology (GO) functional annotation, and the Kyoto Encyclopedia of Genes and Genomes (KEGG). The corrected *p*-value < 0.05 was considered statistically significant.

### 4.6. Quantitative RT-PCR Validation

The samples subjected to qRT-PCR originated from RNA in transcriptome sequencing. Randomly selected 15 DEGs were quantitatively validated for their expression levels through qRT-PCR. All of the primers were designed using Primer 6.0 software (Premier, Ottawa, ON, Canada) (Table 4). The β-tubulin gene (Forward/Reverse primers, AGGCTTTCTTGCATTGGTACACGC/TATTCGCCTTCTTCCTCATCGGCA) was used as an endogenous control for normalization. Quantitative real-time PCR was carried out utilizing a CFX96 real-time PCR detection system. The composition of the qRT-PCR reaction mixture was meticulously prepared: 10 μL 2 × Taq Pro Universal SYBR qPCR Master Mix (Vazyme Biotech, Nanjing, China); 0.4 μL primers for each; 8.2 μL ddH_2_O, 1 μL cDNA, and the final volume was 20 μL. The reaction was performed on a qRT-PCR instrument using the following cycling parameters: 95 °C 120 s; followed by 39 cycles of 95 °C 5 s and 60 °C 30 s. Expression fold changes of the fifteen DEGs were calculated by adopting the 2^−ΔΔCt^ method, with β-tubulin serving as the internal reference genes. Each qRT-PCR run incorporated triplicates for enhanced reliability.

### 4.7. Measurement of Enzyme Activity

Laccase activity was assessed utilizing 2,2′-azino-bis (3-ethylbenzthiazoline-6-sulphonate) (ABTS). A 3 mL reaction mixture was prepared, including 1 mL ABTS (1 M), 1.9 mL citric acid sodium-hydrogen-phosphate buffer (0.1 M, pH 5.0), and 0.1 mL supernatant. The progression of ABTS oxidation was monitored through an absorbance increase at 420 nm (ε = 36,000 M^−1^ cm^−1^). One unit of enzyme activity was established as the catalytic conversion of 1 μmol of ABTS within a minute [65]. CMCase activity determination was executed using the 3,5-dinitrosalicylic acid (DNS) method at 540 nm, with one unit established as the amount of enzyme releasing 1 µmol of reducing sugars equivalent to CMCase within a minute [66]. Chitinase activity measurement was carried out colorimetrically, employing colloidal chitin as the substrate. One unit of chitinase activity was established as the amount of enzyme producing 1 μmol of N-acetyl glucosamine under 37 °C within a minute [67]. Amylase activity assessment relied on the DNS method to quantify reducing sugars released from starch. Here, one unit of amylase activity was established as the quantity of enzyme required to produce 1 μmol of reducing sugar within a minute [68].

## 5. Conclusions

In this study, RNA-seq was used for the first time to investigate the response of *P. ostreatus* to OMSV infection. A total of 354 DEGs were identified and analyzed. Both GO and KEGG analyses highlighted the pivotal role of primary metabolic pathways in OMSV infection. Also, the activities of extracellular enzymes, including laccase, CMCase, amylase, and chitinase, were also analyzed during OMSV infection in *P. ostreatus* to examine the relationship between OMSV infection and the phenotypic changes it induced in *P. ostreatus*. This study offered a theoretical basis for deeper exploration of the pathogenic mechanisms of OMSV in *P. ostreatus*, thus assisting in developing disease-resistant varieties of edible fungi and preventing and controlling diseases.

## Figures and Tables

**Table 1:** Reads in the reference genome alignment results.

Samples	Clean Reads	Clean Bases	GC Content	% ≥ Q30
Mock-1	20,965,286	6,276,707,342	53.67%	92.68%
Mock-2	23,424,577	7,016,262,210	53.80%	94.51%
Mock-3	22,083,023	6,613,712,742	53.68%	92.63%
OMSV-1	24,515,659	7,343,439,530	54.18%	94.47%
OMSV-2	24,050,037	7,202,732,604	54.08%	94.54%
OMSV-3	22,548,293	6,753,639,986	54.24%	94.88%

**Table 2:** The number of DEGs in the top 10 KEGG pathways.

Pathway	DEGs with Pathway Annotation	Pathway ID	*p*-Value
Glyoxylate and dicarboxylate metabolism	6 (6.12%)	ko00630	0.000953
Glycine, serine, and threonine metabolism	9 (9.18%)	ko00260	0.000997
Fatty acid biosynthesis	5 (5.10%)	ko00061	0.003113
Penicillin and cephalosporin biosynthesis	2 (2.04%)	ko00311	0.005509
D-Arginine and D-ornithine metabolism	2 (2.04%)	ko00472	0.005509
Propanoate metabolism	5 (5.10%)	ko00640	0.008949
One carbon pool by folate	3 (3.06%)	ko00670	0.010096
Phenylalanine, tyrosine, and tryptophan biosynthesis	3 (3.06%)	ko00400	0.014425
Non-homologous end-joining	3 (3.06%)	ko03450	0.014425
Cutin, suberine, and wax biosynthesis	2 (2.04%)	ko00073	0.018137

**Table 3:** The 15 DEGs verified by qRT-PCR.

Genes	Annotation	log2FoldChange	2^−ΔΔCt^
g1073569	Methylenetetrahydrofolate dehydrogenase	−1.92	0.97
g1052256	S-adenosylmethionine synthetase	−1.48	0.34
g1047319	Polyketide synthase	−3.64	0.80
g1073665	Aryl-alcohol dehydrogenase	−1.93	0.05
g1088328	Enoyl-(Acyl carrier protein) reductase	1.70	2.16
g1113424	Cytochrome P450	−2.14	0.18
g1074848	Chitinase	1.75	1.32
g1077485	Nicotinate-nucleotide pyrophosphorylase	−0.70	0.85
g1088664	1,3-beta-glucosidase	0.66	1.26
g1103527	Beta-glucan synthesis-associated protein	−0.82	0.64
g1036297	Polysaccharide lyase family 8 protein	1.03	2.29
g51725	Laccase	−0.60	0.77
g1067151	Chitin synthase	−2.41	0.09
g1044280	Lipase	1.96	1.06
g1046093	50S ribosome-binding GTPase	3.50	5.81

**Table 4:** Candidate gene primer sequences.

Gene ID	Forward Primer (5′→3′)	Reverse Primer (5′→3′)	Size (bp)
g1073569	GAAGTCGGAGGCCGTATCAG	CATACGTCGAGGAATCGGGG	103
g1052256	TGGTCACCACTGTCGTCTTG	CGATGCGTGTAGAAGGTGGT	114
g1047319	TGACCGCTCGCAGACAAAT	CTTCTCGATAAGCTCAGCCTTGG	74
g1088328	TTGTTACCGGCCAGGTGATG	AAACATCGAGTTTGCATCGCT	76
g1044280	TTTCTGGGTTTCCACCACCC	TCTTGGCCTGGACAGGAAAC	80
g1073665	ACAGTTCATCGGTGAGTGGG	GCCGTGTACTTGGTCGAGATA	72
g1067151	GGGAGCTGCTTTGGATGAGT	ACTGTGAGGCACAGCGATAG	86
g1113424	AGTGGCAGGTTCTTGGTCTG	CAAAGGTAAGGCGAACGACG	115
g1046093	TGGATCTTCCTGAAACCGCC	TAGCACGAGGGAAGAGTTAAGTG	145
g1074848	TTCGGGCACATCTTATTGGGG	ATCGATGGAATCGTCCTGGC	106
g1077485	ATGTTCGTCGTGGCACTTCT	CAGCGATATGAGAGTGGCGT	100
g1088664	CCCATCGTCTCAGCCTTCAA	ACTCTACTCACGGTCTGCGA	91
g1103527	ACCTCGGTTTCTCTCCCAGG	GCTCTGCCGAGATTACCCAT	80
g1036297	TGGTACACCGTGGACGCAAT	CATTCTGTGTTGACGGTGCG	70
g51725	GGATGGATACAGTCGCCAGG	CAATCTGAAGGTGTCGCCCT	88

## Data Availability

The raw RNA-seq data of the present study were deposited into the NCBI database with an accession number of PRJNA1150065.
